# 3D Reconstruction and Holographic XR Navigation in Robot‐Assisted Sacrocolpopexy: A Report of Two Cases

**DOI:** 10.1111/jog.70421

**Published:** 2026-07-27

**Authors:** Tsukuru Amano, Daiki Idegami, Yutaka Yoneoka, Atsushi Murakami, Yuji Tanaka, Hiroyuki Yamanaka, Akimasa Takahashi, Shunichiro Tsuji

**Affiliations:** ^1^ Department of Obstetrics and Gynecology Shiga University of Medical Science Seta, Otsu Japan

**Keywords:** holographic visualization, image‐guided surgery, pelvic organ prolapse, robot‐assisted sacrocolpopexy, three‐dimensional reconstruction

## Abstract

Dissection at the sacral promontory during robot‐assisted sacrocolpopexy (RSC) is technically demanding because of the proximity of major vascular structures and anatomical variability. We report two cases of RSC in which preoperative three‐dimensional (3D) reconstructed imaging and holographic extended reality (XR) visualization were used for surgical planning and intraoperative navigation. In Case 1, 3D reconstructed imaging demonstrated a narrow vascular window anterior to L5, and mesh fixation was performed on the anterior longitudinal ligament over S1 under image‐guided navigation. In Case 2, holographic XR visualization enabled preoperative distance assessment and identification of a vascularly safe region extending approximately 1.7 cm cranial to the sacral promontory, which was subsequently reproduced intraoperatively using the TilePro function on the da Vinci surgeon console. Both procedures were completed without vascular injury. Preoperative 3D reconstruction and holographic XR visualization may facilitate safer sacral promontory dissection during RSC by improving spatial understanding of patient‐specific vascular anatomy and supporting patient‐specific surgical planning and intraoperative navigation.

## 1 Introduction

Pelvic organ prolapse (POP) is a common condition that significantly impairs women's quality of life. Robot‐assisted sacrocolpopexy (RSC) has become a widely accepted and effective surgical procedure for the treatment of apical prolapse, providing durable anatomical outcomes with reduced perioperative morbidity compared with open surgery [1]. Despite these advantages, RSC includes critical steps that carry a risk of serious complications. In particular, dissection and fixation at the sacral promontory are technically demanding because of the close proximity of major vascular structures, including the common iliac vessels and the presacral venous plexus, as well as adjacent nerves and ureters, and may result in life‐threatening complications [2, 3]. Vascular injury in this setting can lead to massive hemorrhage and represents one of the most severe complications in minimally invasive pelvic floor reconstructive surgery. Among these vessels, the left common iliac vein is particularly vulnerable because it courses near the midline with considerable anatomical variation and may lie within 1 cm of the midline, posing a high risk of inadvertent injury [4]. Recent studies using three‐dimensional computed tomography angiography (3DCTA) have demonstrated that anatomical variations of the iliac veins are common and can significantly restrict the vascular window for safe dissection at the sacral promontory. However, although these findings highlight the importance of preoperative anatomical assessment, the integration of such information into intraoperative navigation remains limited [5]. Therefore, precise preoperative identification of patient‐specific vascular anatomy and its translation into effective intraoperative navigation are essential to minimize the risk of vascular injury.

Recent advances in imaging technologies, particularly three‐dimensional (3D) reconstruction, have enabled detailed visualization of individualized anatomy. In addition, extended reality (XR) technologies have further expanded these capabilities by enhancing spatial understanding [6]. Together, these approaches may help bridge the gap between preoperative imaging and intraoperative decision‐making during RSC.

In this report, we describe two cases of RSC for POP in which preoperative 3D reconstructed imaging and XR were used to evaluate vascular anatomy around the sacral promontory. These technologies provided patient‐specific anatomical information that informed surgical strategy and facilitated intraoperative navigation during sacral promontory dissection.

## 2 Case Presentation

### 2.1 Case 1

**FIGURE 1:**
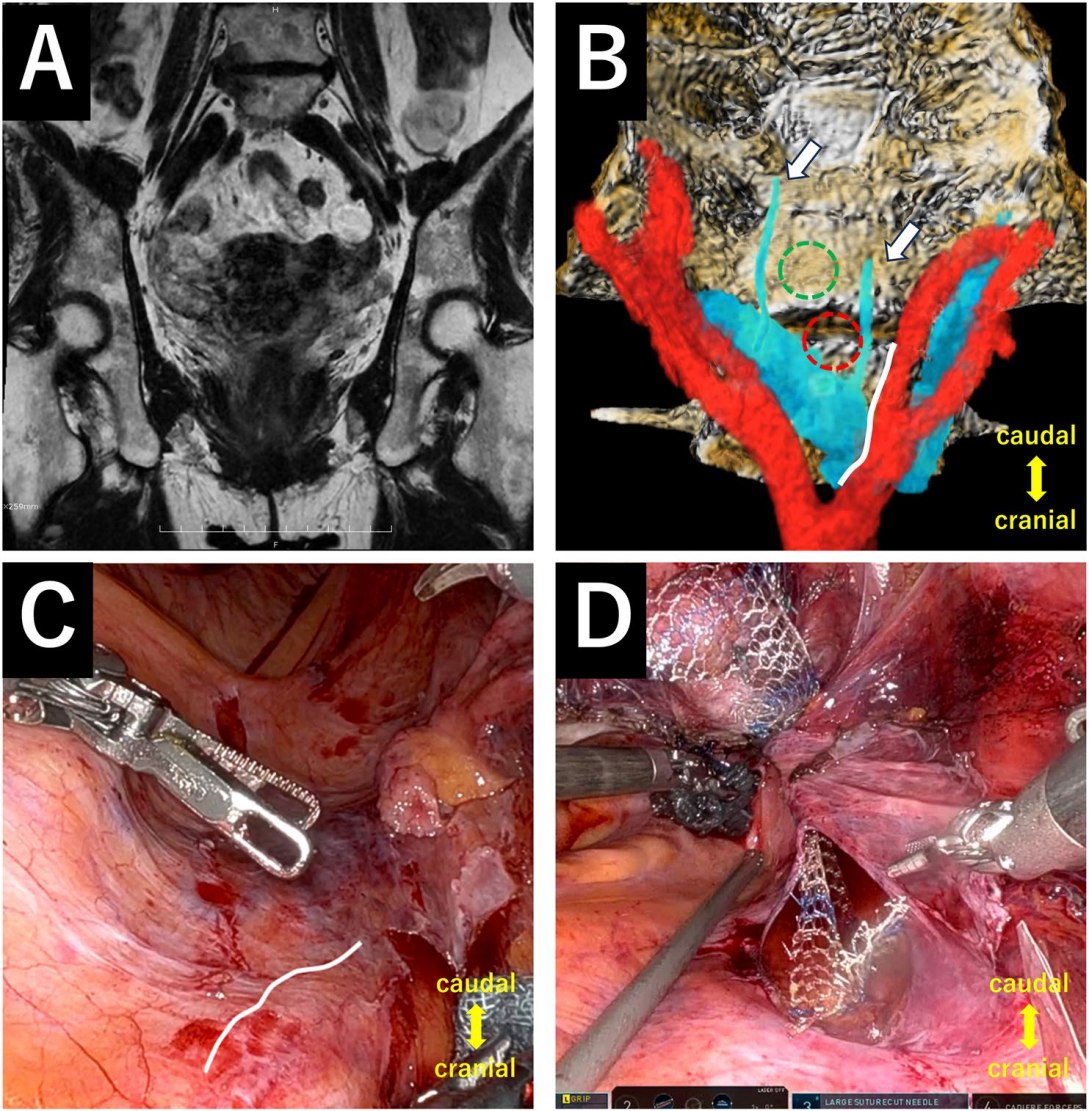
Preoperative three‐dimensional (3D) reconstructed imaging and intraoperative navigation in Case 1. (A) Coronal T2‐weighted magnetic resonance imaging. (B) Preoperative 3D reconstructed imaging generated using the SYNAPSE VINCENT system demonstrating vascular anatomy around the sacral promontory. Arterial structures are shown in red and venous structures in blue. The yellow circle indicates the narrow vascular window identified on preoperative 3D reconstruction. The green circle indicates the selected fixation site on the anterior surface of S1. White arrows indicate venous branches running along the anterior sacral surface, which were also considered during preoperative surgical planning. The white line indicates the medial border of the right common iliac artery continuing to the internal iliac artery. (C) Intraoperative view during sacral promontory dissection. The right common iliac artery was identified by arterial pulsation and gentle palpation using the robotic forceps. This vessel served as the anatomical landmark for correlating the preoperative 3D reconstructed image with the operative field. (D) Intraoperative findings during robot‐assisted sacrocolpopexy with mesh fixation on the anterior longitudinal ligament over S1.

A 72‐year‐old multiparous woman (gravida 3, para 2) presented with symptomatic POP. She had been managed with a pessary ring for 13 years but experienced progressive symptoms requiring surgical intervention. Coronal T2‐weighted magnetic resonance imaging was acquired without contrast enhancement, with a slice thickness of 1.6 mm and an in‐plane spatial resolution of 0.8 mm (Figure 1A). The imaging data were imported into the SYNAPSE VINCENT system (Fujifilm, Tokyo, Japan) for 3D reconstruction and preoperative anatomical assessment. Using these reconstructed images, vascular anatomy around the sacral promontory was evaluated preoperatively (Figure 1B). During surgery, the 3D reconstructed images were displayed on an operating room monitor and used for intraoperative anatomical navigation. Because the sacral promontory was relatively less distinct intraoperatively owing to the relatively shallow lumbosacral angle, the medial border extending from the right common iliac artery to the right internal iliac artery was used as the anatomical landmark (Figure 1C). This vascular line was then confirmed by arterial pulsation and gentle palpation using the robotic forceps. This vascular line served as the anatomical landmark for correlating the preoperative 3D reconstructed images with the operative field, enabling recognition of the surrounding vascular anatomy and a safe dissection plane. Based on these patient‐specific anatomical findings, RSC was performed under image‐guided navigation. Preoperative evaluation confirmed that the vascular window anterior to L5 was extremely narrow because of the close proximity of the left common iliac vein. Therefore, fixation cranial to the sacral promontory was avoided, and the mesh was fixed to the anterior surface of S1 (Figure 1D). During dissection over the anterior aspect of S1, the reconstructed imaging was also used to identify a safe dissection plane and avoid injury to venous branches. The procedure was completed without vascular injury. Operative time was 211 min, and estimated blood loss was 0 mL. The postoperative course was uneventful, and the patient was discharged on postoperative day 5.

### 2.2 Case 2

**FIGURE 2:**
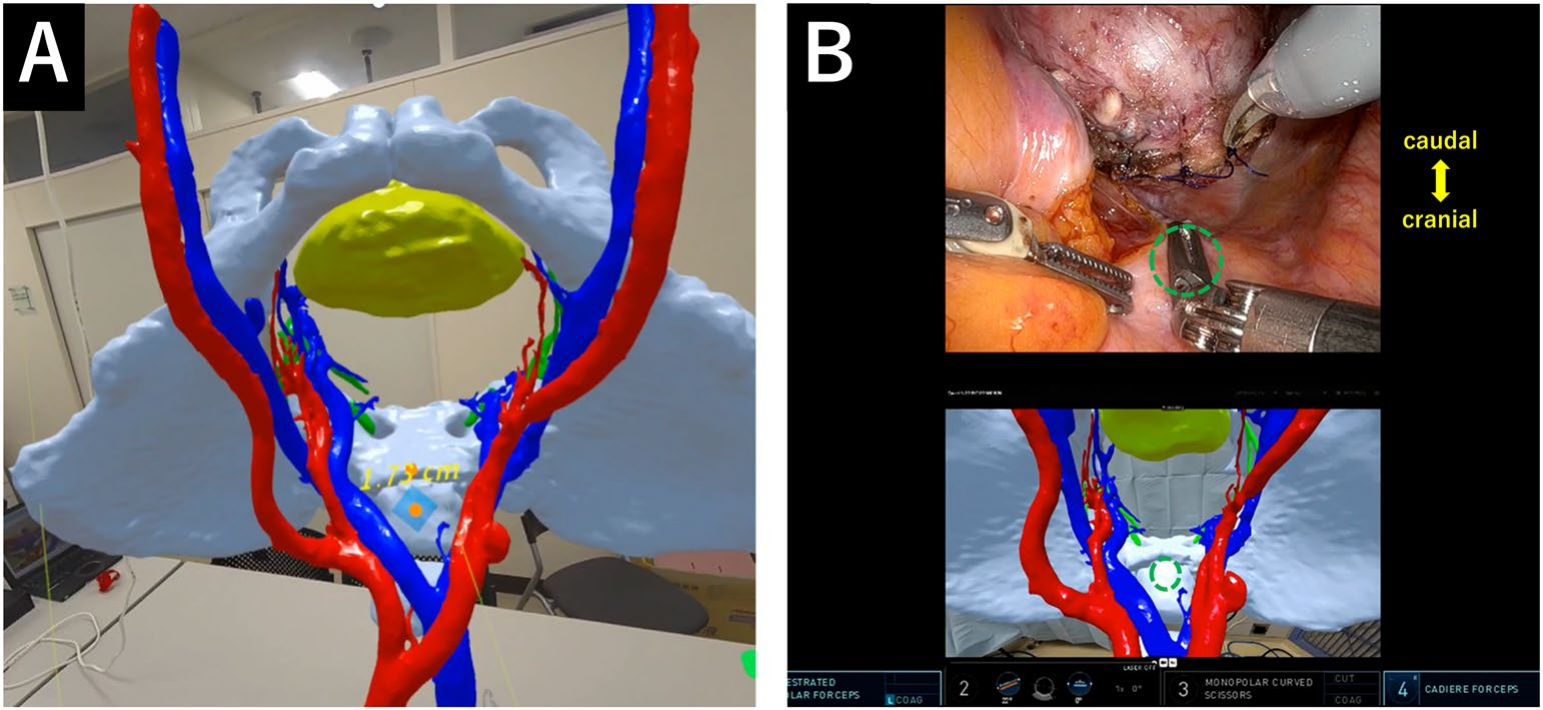
Holographic visualization and intraoperative navigation in Case 2. (A) Holographic visualization generated from patient‐specific three‐dimensional reconstructed imaging using the Holoeyes platform. Arterial structures are shown in red, venous structures in blue, and neural structures in green. The holographic model enabled preoperative assessment of the anatomical relationship between major vascular structures and the planned fixation area. (B) Intraoperative use of the TilePro system displaying the operative field and holographic visualization simultaneously. Cranial and caudal orientation markers are shown. The green circle indicates the vascularly safe zone extending approximately 1.7 cm cranial to the sacral promontory that was identified preoperatively and used as an anatomical reference during intraoperative navigation.

A 75‐years‐old multiparous woman (gravida 4, para 4) presented with POP. She had been managed with a pessary ring for 8 years; however, progressive vaginal wall erosion made continued pessary management difficult. Because she desired surgical treatment, she was referred to our department from a local clinic. The same MRI acquisition protocol as in Case 1 was used, consisting of non‐contrast coronal T2‐weighted imaging with a slice thickness of 1.6 mm and an in‐plane spatial resolution of 0.8 mm. The imaging data were imported into the SYNAPSE VINCENT system for 3D reconstruction. STL data generated from the reconstructed images were subsequently transferred to the Holoeyes platform (Holoeyes Inc., Tokyo, Japan) to create holographic visualizations of the patient‐specific anatomy (Figure 2A, Video S1). Because severe diabetic retinopathy with unilateral blindness limited the use of steep Trendelenburg positioning, fixation cranial to the sacral promontory was planned preoperatively to facilitate exposure of the anterior longitudinal ligament. Holographic visualization enabled accurate spatial recognition and distance assessment, demonstrating that the region extending approximately 1.7 cm cranial to the sacral promontory was free of major vascular structures. This information facilitated preoperative planning by confirming the presence of a vascularly safe region, allowing fixation slightly cranial to the sacral promontory while maintaining an appropriate relationship with adjacent vascular structures. During surgery, the holographic images were displayed on the surgeon console of the da Vinci surgical system using the TilePro function, allowing real‐time intraoperative reference to the vascular anatomy around the sacral promontory (Figure 2B). The previously identified vascularly safe region was reproduced intraoperatively by referring to the holographic image displayed on the surgeon console and using the known length of the da Vinci instrument tip as a spatial reference (Video S2). This enabled accurate identification of the planned fixation area while maintaining awareness of the surrounding vascular anatomy. RSC was successfully completed without intraoperative vascular complications. Operative time was 189 min, and estimated blood loss was 0 mL. The postoperative course was uneventful, and the patient was discharged on postoperative day 3.

## 3 Discussion

In this report, we demonstrated that the combination of preoperative 3D imaging and intraoperative visualization techniques may serve as a useful tool for safer sacral promontory dissection during RSC. In Case 1, 3D reconstruction demonstrated a markedly restricted vascular window anterior to L5 caused by the close proximity of the left common iliac vein. Based on these findings, fixation cranial to the sacral promontory was avoided, and fixation on the anterior surface of S1 was selected as a safer alternative. Case 2 demonstrated the feasibility of integrating holographic XR visualization into preoperative simulation and intraoperative navigation.

Image‐guided surgery has increasingly been explored across various surgical fields and has demonstrated potential benefits in improving anatomical orientation and reducing complications [7]. Similar technologies have been increasingly applied in robotic and minimally invasive surgery. In urologic surgery, particularly robot‐assisted partial nephrectomy, augmented reality and holographic imaging have been used for visualization of tumor–vascular relationships and intraoperative navigation [8, 9]. Comparable applications have also been reported in hepatobiliary surgery for preoperative planning, tumor localization, intraoperative navigation, and liver resection guidance [10, 11, 12]. In gynecologic surgery, 3D image‐guided navigation has also been applied to sentinel lymph node biopsy in endometrial cancer surgery, supporting the feasibility of this approach in the pelvic surgical field [13]. Overall, image‐guided navigation technologies may be particularly well suited to procedures involving relatively fixed anatomical structures with predictable spatial relationships. In gynecologic surgery, endometrial cancer sentinel lymph node biopsy and sacral promontory dissection during RSC represent typical examples of such procedures, in which image‐guided navigation may facilitate anatomical orientation and improve surgical safety. In RSC, the anatomical relationship between the sacral bone and the adjacent major vessels is relatively constant; therefore, image‐guided navigation may be useful for facilitating safer sacral promontory dissection. In addition to the left common iliac vein, injury to the median sacral vessels is another potential source of bleeding during sacral promontory dissection. Although visualization of these vessels may depend on vessel caliber and image quality, larger median sacral vessels may be identifiable on preoperative 3D reconstruction and should be considered during surgical planning.

Whereas Case 1 utilized conventional 3D reconstructed images displayed on an operating room monitor, Case 2 incorporated holographic XR visualization for both preoperative simulation and intraoperative navigation. Although Case 2 did not exhibit a particularly hazardous vascular configuration, holographic XR visualization provided depth perception, enabling a more intuitive understanding of the 3D spatial relationship between the sacral promontory and adjacent vascular structures. It also facilitated preoperative distance assessment and identification of a vascularly safe region extending approximately 1.7 cm cranial to the sacral promontory. Integration of holographic images into the surgeon console through the TilePro system provided an intraoperative anatomical reference while reducing the need for repeated visual attention shifts between the operative field and external monitors. Because the holographic image reproduced the operative orientation of the sacral promontory, including the angle between L5 and S1, the preoperatively identified vascularly safe region could be reproduced intraoperatively using the known length of the da Vinci instrument tip as a spatial reference, allowing accurate mesh fixation while maintaining awareness of the surrounding vascular anatomy. Similar advantages of XR‐assisted visualization have been reported in hepatobiliary and thoracic surgery [14, 15].

Several limitations of this technology should be acknowledged. Because venous structures may collapse under pneumoperitoneum, they may appear flatter than on preoperative 3D reconstructed images, and this possibility should be taken into consideration. In addition, integration of holographic XR images into the surgeon console reduces the display area available for the actual surgical view. Although experienced pelvic surgeons can generally perform sacral promontory dissection safely using conventional anatomical landmarks, XR‐assisted navigation may provide additional support by facilitating patient‐specific anatomical understanding and spatial orientation. Nevertheless, the incremental clinical value of XR‐assisted navigation over conventional techniques remains to be established through future prospective studies.

In conclusion, preoperative 3D reconstruction and holographic XR visualization may contribute to safer sacral promontory dissection during RSC by facilitating patient‐specific surgical planning, spatial understanding of anatomy, and intraoperative navigation.

## Author Contributions

**Daiki Idegami:** data curation, software, validation, visualization. **Hiroyuki Yamanaka:** investigation. **Akimasa Takahashi:** investigation. **Yuji Tanaka:** investigation. **Tsukuru Amano:** writing – original draft, conceptualization, project administration, methodology, resources, investigation. **Shunichiro Tsuji:** writing – review and editing, supervision. **Yutaka Yoneoka:** formal analysis. **Atsushi Murakami:** investigation, resources, software.

## Funding

The authors have nothing to report.

## Ethics Statement

Institutional review board approval was waived because this study describes a retrospective presentation of anonymized clinical cases.

## Consent

Written informed consent was obtained from the patients for publication of this case report and accompanying images.

## Conflicts of Interest

The authors declare no conflicts of interest.

## Supporting information


**Video S1:** Holographic visualization of patient‐specific vascular anatomy in Case 2. Holographic XR images generated from three‐dimensional reconstructed imaging using the Holoeyes platform are shown. Arterial structures are shown in red, venous structures in blue, and neural structures in green. The holographic model enabled intuitive spatial understanding of the anatomical relationship between the sacral promontory, surrounding vascular structures, and the planned fixation area. It also allowed preoperative distance assessment and surgical planning.

**Video S2:** Intraoperative navigation using holographic visualization during robot‐assisted sacrocolpopexy in Case 2. Holographic XR images generated from patient‐specific 3D reconstructed imaging are displayed on the da Vinci surgeon console using the TilePro function during sacral promontory dissection and mesh fixation. Arterial structures are shown in red, venous structures in blue, and neural structures in green. This video demonstrates the feasibility of integrating holographic XR visualization into intraoperative navigation during robot‐assisted sacrocolpopexy.

## Data Availability

The data that supports the findings of this study are available in the Supporting Information of this article.
